# The Modular Circuitry of Apicomplexan Cell Division Plasticity

**DOI:** 10.3389/fcimb.2021.670049

**Published:** 2021-04-12

**Authors:** Marc-Jan Gubbels, Isabelle Coppens, Kourosh Zarringhalam, Manoj T. Duraisingh, Klemens Engelberg

**Affiliations:** ^1^ Department of Biology, Boston College, Chestnut Hill, MA, United States; ^2^ Department of Molecular Microbiology and Immunology, Bloomberg School of Public Health, Johns Hopkins University, Baltimore, MD, United States; ^3^ Department of Mathematics, University of Massachusetts Boston, Boston, MA, United States; ^4^ Department of Immunology and Infectious Diseases, Harvard T. H. Chan School of Public Health, Boston, MA, United States

**Keywords:** Apicomplexa, cell division, cell cycle, karyokinesis, schizogony, endodyogeny, endopolygeny, binary fission

## Abstract

The close-knit group of apicomplexan parasites displays a wide variety of cell division modes, which differ between parasites as well as between different life stages within a single parasite species. The beginning and endpoint of the asexual replication cycles is a ‘zoite’ harboring the defining apical organelles required for host cell invasion. However, the number of zoites produced per division round varies dramatically and can unfold in several different ways. This plasticity of the cell division cycle originates from a combination of hard-wired developmental programs modulated by environmental triggers. Although the environmental triggers and sensors differ between species and developmental stages, widely conserved secondary messengers mediate the signal transduction pathways. These environmental and genetic input integrate in division-mode specific chromosome organization and chromatin modifications that set the stage for each division mode. Cell cycle progression is conveyed by a smorgasbord of positively and negatively acting transcription factors, often acting in concert with epigenetic reader complexes, that can vary dramatically between species as well as division modes. A unique set of cell cycle regulators with spatially distinct localization patterns insert discrete check points which permit individual control and can uncouple general cell cycle progression from nuclear amplification. Clusters of expressed genes are grouped into four functional modules seen in all division modes: 1. mother cytoskeleton disassembly; 2. DNA replication and segregation (D&S); 3. karyokinesis; 4. zoite assembly. A plug-and-play strategy results in the variety of extant division modes. The timing of mother cytoskeleton disassembly is hard-wired at the species level for asexual division modes: it is either the first step, or it is the last step. In the former scenario zoite assembly occurs at the plasma membrane (external budding), and in the latter scenario zoites are assembled in the cytoplasm (internal budding). The number of times each other module is repeated can vary regardless of this first decision, and defines the modes of cell division: schizogony, binary fission, endodyogeny, endopolygeny.

## Introduction

**Figure 1:**
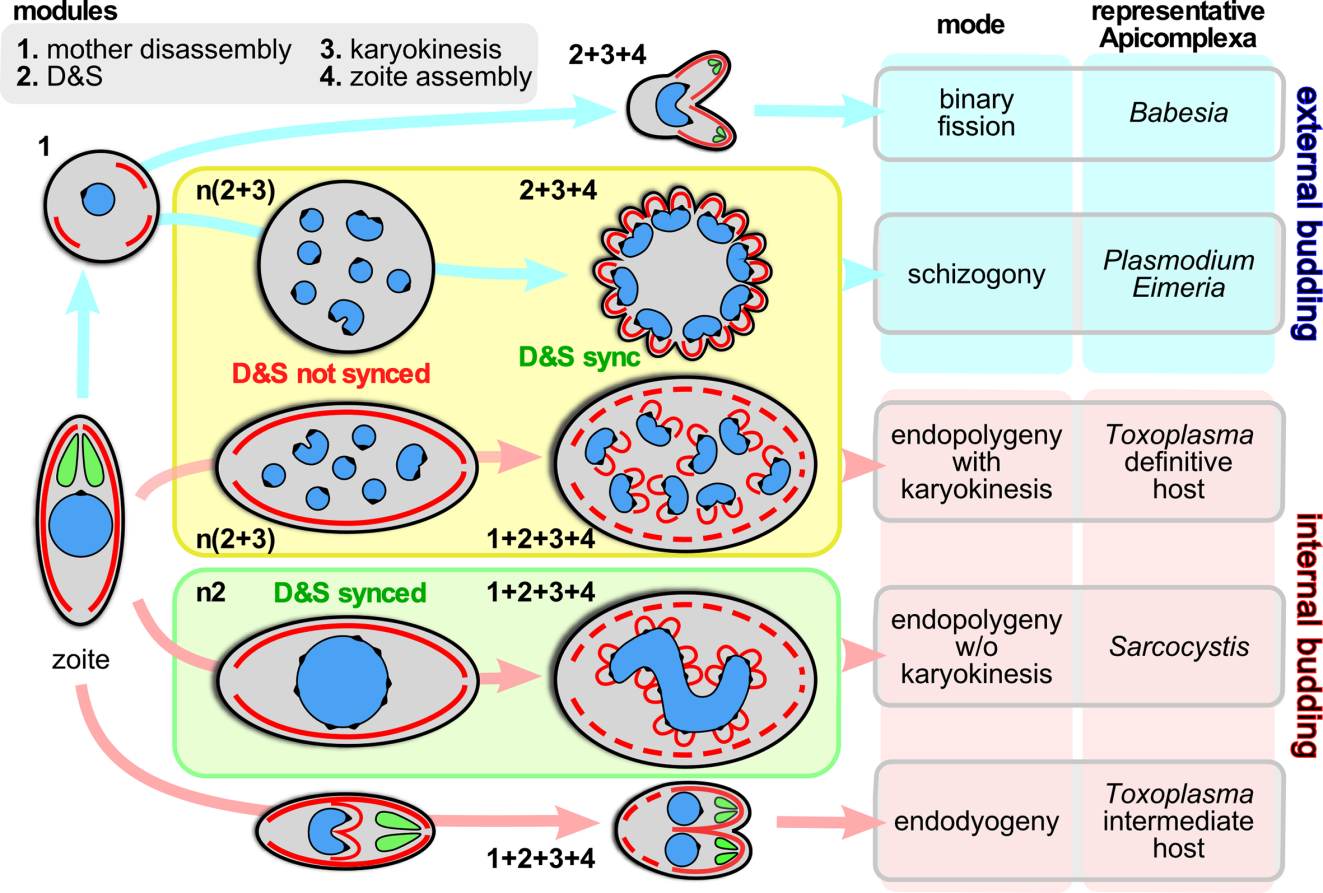
Division modes are composed of modular programs. Schematic representation of the asexual division modes across the Apicomplexa differentiated by external budding and internal budding organized in program modules. Within the yellow box are the division modes wherein D&S is followed by karyokinesis; within the green box the division mode wherein D&S is not followed by karyokinesis (not depicted is the variation of schizogony where at a low frequency the karyokinesis step is skipped following D&S, which occurs during sporogony of *Plasmodium* in the mosquito midgut (Simonetti, 1996) and in the tick salivary gland for most piroplasms, including *Theileria* spp. and many *Babesia* spp. (Mehlhorn and Shein, 1984; Jalovecka et al., 2018). The numbers next to each schematic represent the modules being executed, where “n” indicates the modules are repeated. “D&S” refers to DNA replication and segregation. Parasite structures are as follows: blue, nucleus; black spot on the nucleus; centromere cluster (at the centrocone); red, IMC/cortical cytoskeleton; green, rhoptries (as representative of the apical secretory organelles).

The phylum Apicomplexa harbors a staggering diversity of asexual cell division modes (Gubbels et al., 2020). All division modes have the same beginning- and end-point: an invasion competent ‘zoite’ harboring the apical complex composed of secretory organelles and cytoskeletal elements. Zoite formation progresses with the formation of buds in an apical to basal direction around the centrosome, which in turn is anchored to the nucleus. For clarity, we define the events between going from 1 mother zoite to the emergence of daughter zoites, whether they number only 2 or 10,000s, as a single division round. Besides the number of zoites per division round, the other major variations in cell division are defined by whether daughter budding takes place in the cytoplasm or at the plasma membrane (**Figure 1**), whether each round of DNA replication and segregation is followed by karyokinesis, and whether the mother’s cytoskeleton is disassembled first or last in the process (**Figure 1**).

Here we focus on the how the diversity in asexual division modes across different species as well as between different development stages is organized, how and where the decisions toward which division modes and their progression are made, and how they are transduced toward specific transcriptional profiles. We are considering five archetypical division modes in this review in a number of representative species (**Figure 1**): schizogony in *Plasmodium* spp.; binary fission in the large *Babesia* spp.; endodyogeny in *Toxoplasma gondii* tachyzoites; endopolygeny with karyokinesis in *Cystoisospora suis* and *T. gondii* merozoites; endopolygeny without karyokinesis in *Sarcocystis neurona* (Gubbels et al., 2020). These all represent parasites with an impact on humans either as direct pathogens (*Plasmodium, Babesia, Toxoplasma*) or pathogens of animals relevant to humans (*Cystoisospora, Sarcocystis, Babesia*). This selection does by no means exhaust the diversity observed across the Apicomplexa but it represents the species whose division modes have been studied to a reasonable extent at the ultrastructural, cell biological, and molecular level.

To unravel the rules and principles underlying this variation in division rounds we first defined a series of discrete events, termed modules, that can be strung together in different orders, combinations and/or repeated into the various division modes. We have identified four different modules: 1. mother cytoskeleton disassembly; 2. DNA replication and segregation (D&S); 3. karyokinesis; 4. zoite assembly (daughter budding) (**Figure 1**). A combination of genetically defined developmental and/or environmental cues govern when each division mode will be executed and how many rounds of each module (combination) will be repeated until zoite assembly is initiated.

In this review we present the nature of the modules one at a time followed by an overview of the insights regarding the hierarchy of signaling events and cell cycle controls underlying the specific module sequences and/or repeats. The division modules might be wired differently in the sexual stages, where the general rules laid out here for the asexual stages are in many cases not applicable, which obviously only adds to the complexity and plasticity of the regulating circuitry needed to execute all these division modes.

## Material and Methods

### Immunofluorescence Assays and Expansion Microscopy

RH strain *T. gondii* was maintained in human foreskin fibroblasts (HFF) or hTERT immortalized HFF cells as previously described (Roos et al., 1994). *T. gondii* tachyzoites expressing YFP-tagged IMC3 (Gubbels et al., 2004) were methanol fixed and co-stained with rabbit α-GAP45 [kindly provided by Dr. Con Beckers; (Gaskins et al., 2004)] and DAPI.

Expansion microscopy (ExM) of *Toxoplasma* tachyzoites was achieved by following recently published protocols (Gambarotto et al., 2019; Le Guennec et al., 2020; Tosetti et al., 2020). Briefly, tachyzoites growing in HFFs for ~20 hrs were fixed with -20°C methanol for 7 mins and incubated in 2xsolution (2% formaldehyde, 1.4% acrylamide (AA) in PBS) for five hours at 37°C. Gelation was done in monomer solution (19% (w/w) sodium acrylate, 10% (w/w) AA and 0.1% (w/w) BIS-AA in PBS) complemented with APS and TEMED for 1 hr at 37°C, followed by incubation in denaturation buffer (200 mM SDS, 200 mM NaCl, 50 mM Tris, pH9) at 95°C for 90 mins. Gels were incubated for a first round of expansion in ddH_2_O overnight and washed twice in PBS the next morning. As a primary antibody, rabbit α-Tgβ-tubulin (kindly provided by Dr. Naomi Morrissette, University of California, Irvine (Morrissette and Sibley, 2002)) was used to stain parasite microtubules. Gels were incubated in 2% BSA in PBS with primary antibody at 37°C for three hours, washed three times with PBST (1xPBS + 0.1% Tween20) and incubated for three hours at 37°C in 2% BSA in PBS complemented with secondary antibody (goat anti-rabbit-A594, Invitrogen). Gels were washed three times in PBST before a second expansion in ddH_2_O was undertaken overnight. For imaging, gels were mounted in 35 mm glass bottom microwell dishes (MatTek) and imaged on a Zeiss LSM880 with Airyscan unit using standard settings for image acquisition and Airyscan deconvolution. All imaging was done in the Boston College Imaging Core with advice of Dr. Bret Judson.

### Transmission Electron Microscopy

Methods as described in (Jayabalasingham et al., 2010). In brief, after isolation from mosquito salivary glands, *P. berghei* sporozoites were maintained under axenic conditions in culture medium/FBS at 37°C for 12 hrs allowing the transformation into trophozoites (the first liver stage). Pellets of converting sporozoites were fixed in 2.5% glutaraldehyde (Electron Microscopy Sciences; EMS, Hatfield, PA) in 0.1 M sodium cacodylate buffer, pH 7.4, for 1 h at room temperature, and processed as described (Nishikawa et al., 2005) before examination with a Philips CM120 Electron Microscope (Eindhoven, The Netherlands) under 80 kV. Electron microscopy.

## Results

### Modules of Apicomplexan Division Modes

The ultimate product across the asexual division modes is uniformly a host cell invasion-competent zoite. The zoite is defined by a set of apical secretory organelles as well at its cortical membrane skeleton, which together comprise the namesake features of the Apicomplexa and function in host cell invasion. Besides the function of the cortical membrane skeleton in invasion, its assembly is a conserved key feature of the apicomplexan cell division process across the various species and division modes. Importantly, no cell division occurs outside of a host cell, since, with very few exceptions, all Apicomplexa are obligate intracellular parasites. Hence, the invasion competence and the asexual division mode driven by cortical cytoskeleton budding are closely interwoven features of these parasites.

The variations in cell division modes break down into modules that can be aligned and stacked in different combinations and/or sequences. We define four main modules (**Figure 1**): 1. mother zoite cytoskeleton disassembly; 2. combined DNA replication and segregation (D&S); 3. karyokinesis, i.e. partitioning chromosome sets and formation of individual nuclei; 4. zoite assembly (daughter cytoskeleton budding). The most defining feature between the different division modes is whether module #1, mother cytoskeleton disassembly, is the first step or the last step in the division process: this differentiates external budding from internal budding (**Figure 1**), respectively (Gubbels et al., 2020). This difference has significant cell biological consequences e.g. whether zoite assembly starts in association with the plasma membrane or not, and whether the mother and daughter cytoskeletons need to be differentially degraded and stabilized. Another notable feature is that if D&S module #2 is not followed by karyokinesis module #3, the D&S cycles of nuclei in the same cytoplasm progress asynchronously. Although modules, #2, #3, can be repeated several times and even independently, ultimately, to accommodate budding #2, #3 and #4 have to progress sequentially because activation of the zoite assembly module #4 requires a final round of D&S (#2) connected to karyokinesis (#3). Notably, budding is executed synchronously across all nuclei in the same cytoplasm, which requires a synchronization step of asynchronous nuclear division cycles. Completion of any division strategy therefore (almost) always produces an even number of daughters per division round. These observations define the ground rules along which the various cell division modes unfold, however, the big question is how the decisions on module sequence and repetition are made and executed.

### DNA Replication and Segregation

Apicomplexan genomes range in sizes between 1-100 Mb and organized in 3-14 typical eukaryotic chromosomes with telomeric repeats at the end of each chromosome, with a chromatin defined centromere. In both *T. gondii* (Gissot et al., 2012) and *P. falciparum* (O’donnell et al., 2002) the telomeres cluster together and are anchored on the nuclear envelope. At least during division, the centromeres are equally clustered and anchored on the nuclear periphery (Brooks et al., 2011; Hoeijmakers et al., 2012). However, when not dividing, the centromeres are not clustered in *P. falciparum* sporozoites (Bunnik et al., 2019). Moreover, limited centromere dissociation occurs during interphase of *Plasmodium* nuclei replicating during schizogony in the erythrocyte (Arnot et al., 2011; Gerald et al., 2011; Roques et al., 2019; Zeeshan et al., 2020b). It is not clear how universal this rule is since unclustered centromeres are never observed for *T. gondii* tachyzoites (Brooks et al., 2011; Farrell and Gubbels, 2014; Chen et al., 2015b). Either way, there is a high level of chromosomal organization to maintain heterochromatin structure during D&S (Fraschka et al., 2018; Bunnik et al., 2019).

Chromosomal DNA replication in eukaryotes progresses from the origin recognition complex (ORC), a complex composed of six proteins that binds to replication origins and is essential for the initiation of chromosomal DNA replication. Work from *P. falciparum* has demonstrated that members of the ORC complex indeed function in DNA replication and likely bind to autonomously replicating sequences (ARS)-like sequences as putative origins of replication (Mehra et al., 2005; Gupta et al., 2008; Deshmukh et al., 2015; Agarwal et al., 2017; Matthews et al., 2018). The proliferating-cell-nuclear-antigen (PCNA) studied in *T. gondii* is recruited to puncta in the nucleus, highlighting DNA unwinding at replication forks (Guerini et al., 2005). Although in general the DNA replication machinery appears to be conventionally eukaryotic, *T. gondii* DNA replication pauses when genome duplication is 80% completed and then progresses at a much slower pace to 100% replication, resulting in a bi-modal S-phase (Radke et al., 2001). In *Plasmodium* distinct replication dynamics have also been reported, but whether this represents the same phenomenon is currently not clear (Stanojcic et al., 2017).

**Figure 2:**
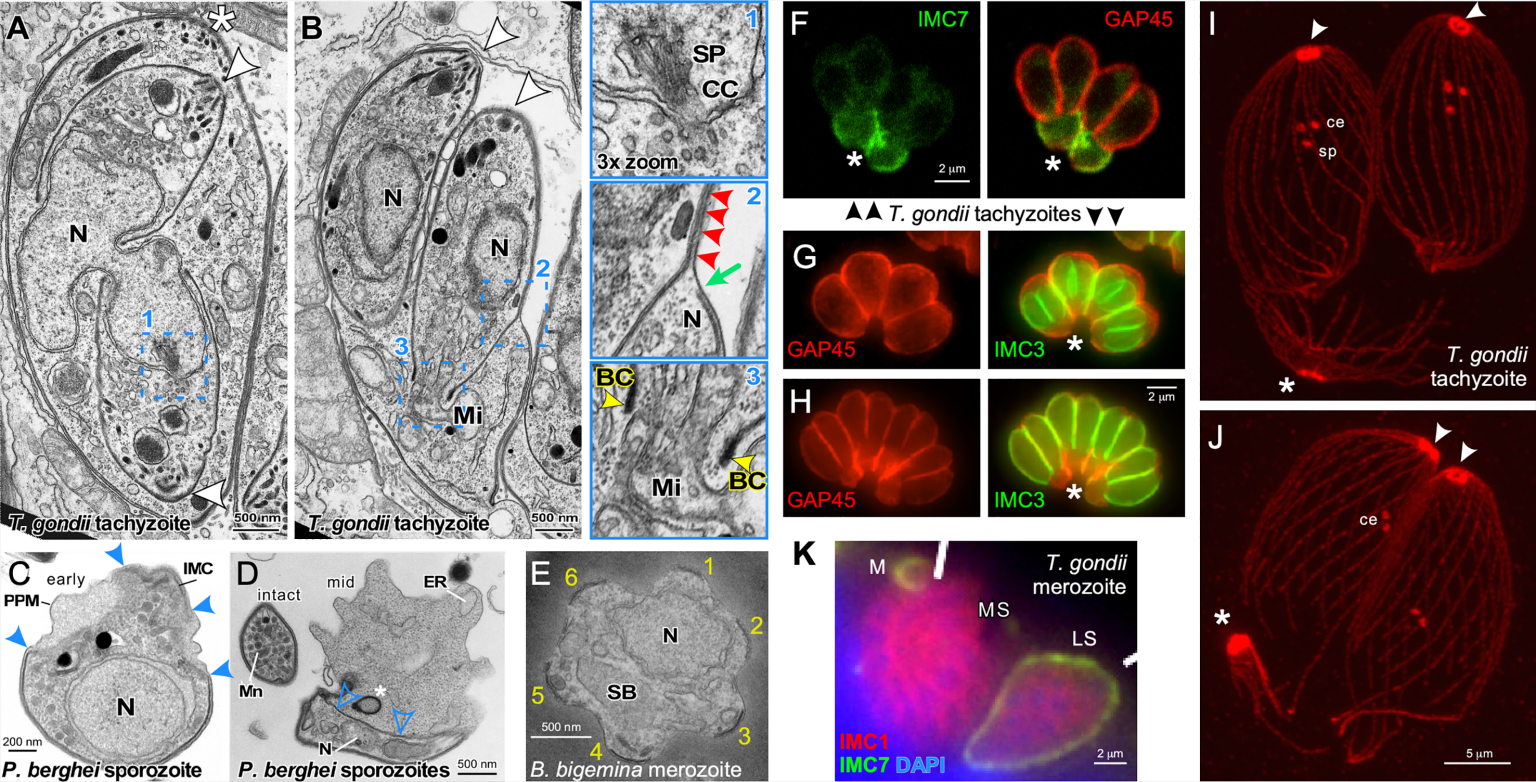
Cytoskeleton disassembly across division modes. **(A, B)** Thin section transmission electron microscopy of *T. gondii* tachyzoites dividing by internal budding through endodyogeny. The parasite in A is undergoing completion of karyokinesis while zoite assembly (daughter budding) progresses with the mother’s cytoskeleton still intact. Half of the spindle (SP) is visible in one of the parasites and terminates in the centrocone (CC), an invagination of the nuclear membrane (enlarged in panel 1). Note the absence of both chromosome condensation and any sign of an electron dense structure potentially driving karyokinesis. Panel **(B)** is at a more advanced division stage with the daughters emerging and the mother’s cytoskeleton being disassembled in apical to basal direction. Note in enlarged panel 2 that only a very small section of plasma membrane is not supported by either mother (green arrow) or daughter IMC (red arrowheads). Enlarged panel 3 highlights the electron dense basal complex (BC; yellow arrowheads) on the extremity of the budding cytoskeleton through which the mitochondrion (Mi) is being partitioned. Note that scission between the two daughter cells involves membrane fusion events to create new plasma membrane. Asterisks mark the apical end of the mother parasites, arrowheads mark the apical ends of the budding daughters. BC, basal complex; N, nucleus. Modified from (Morrissette and Sibley, 2002). **(C, D)** Thin section transmission electron microscopy of *Plasmodium berghei* sporozoites converting to trophozoites in the first steps of external budding by schizogony. The process of conversion is initiated with the dismantling of the IMC that corsets the parasite and the IMC detachment from the parasite plasma membrane, which allows the parasite to expand in size. Solid blue arrowheads mark breakpoints of the IMC. The IMC free in the cytoplasm (open blue arrowheads) undergoes compaction as membrane whorls (asterisk) prior to expulsion from the parasite. PPM, plasma membrane; N, nucleus; Mn, micronemes; ER, endoplasmic reticulum. **(E)** Thin section transmission electron microscopy of a disassembling *Babesia bigemina* merozoite already escaped from the vacuole displaying the typical pattern of six remnants of the disassembling mother IMC (numbered 1-6) as the first steps in external budding by binary fission. N, nucleus; SB, spherical body. Modified from (Gubbels et al., 2020). **(F)** *T. gondii* tachyzoites co-stained with IMC7 and GAP45 antisera in late stages of endodyogeny. IMC7 is only recruited to the cytoskeleton in G1-phase of the cell cycle following completion of cell division. Hence, IMC7 is absent from the emerging daughters marked by GAP45 deposition, while the mother’s IMC is being disassembled in an apical to basal direction as highlighted by IMC7 at the basal ends (asterisk). Differential staining of the mother and daughter cytoskeletons provides a potential mechanism underlying their differential stability. Modified from (Anderson-White et al., 2011). **(G, H)** *T. gondii* tachyzoites expressing YFP-tagged IMC3 in the late stages of endodyogeny co-stained with GAP45 antiserum. The absence of GAP45 co-staining with IMC3 of the daughter marks panel G just before daughter parasites are emerging in the division cycle, whereas robust co-staining in panel H illustrates emerging parasites where GAP45 co-localizes with IMC3. Differential staining of the mother and daughter cytoskeletons provides a potential mechanism underlying their differential stability. Asterisks mark the basal ends of the parasites where GAP45 derived from the mother is accumulating, to be digested in the residual body that is about to form as final remnant of the mother. **(I, J)** Expansion microscopy of *T. gondii* tachyzoites late in cell division stained with β-tubulin antiserum. In panel I the spindle pole (sp) is still visible just below the paired centrioles (ce) in the centrosomes, whereas in panel J the spindle pole has been completely disassembled. Note that the conoid and sub-pellicular microtubules from the mother (asterisks) accumulate at the basal end to be disassembled in the residual body; note disassembly is further progressed in panel J, which is at a more advanced stage in the cell division cycle. **(K)** *T. gondii* merozoites forming by internal budding through endopolygeny co-stained with IMC1 and IMC7 antisera display differential staining of the mother and daughter cytoskeletons, providing a potential mechanism underlying differential stability. IMC7 specifically stains the mother’s mature cytoskeleton, whereas IMC1 is specific to the immature, budding merozoites. M, merozoite; LS, late schizont; MS, mature schizont with nearly emerging merozoites. Modified from (Dubey et al., 2017).

Mitosis has been studied to some extent although many questions remain. The nuclear envelope does not disassemble and only limited chromosome condensation occurs, which is however somewhat variable between parasites and developmental cycle stages. In *Toxoplasma* the set of 13 chromosomes is clustered at their centromeres marked by the variant centromeric histone 3 (a.k.a CENPA) (Brooks et al., 2011). CENPA is associated with the structural maintenance of chromosomes protein 1 (SMC1), which in turn engages TgExportin1, a component of the nuclear pore complex (NPC) (Francia et al., 2020). At the ultrastructural level, the centromere cluster is always present at the nuclear envelope, often in close proximity to an NPC. The association of TgSMC1 with TgExportin1 provides a potential mechanism for this association, although TgExportin localization is not exclusive to the centromeres (Francia et al., 2020). During *Plasmodium* schizogony, the core subunits of condensin, SMC2 and SMC4, transition from a diffuse nuclear pattern to a centromere associated state upon onset of S/M phase, which diffuses again upon completion of the division round (Pandey et al., 2020). Across division modes, the membrane organization and recognition nexus protein 1 (MORN1) is always present at the centromere cluster and marks the specialized NPC, which during mitosis becomes prominently visible as the ‘centrocone’ through which the microtubules penetrate the nuclear envelope (Dubremetz and Elsner, 1979; Gubbels et al., 2006; Ferguson et al., 2008) (**Figure 2A**). During interphase in G1, centromere clustering in *T. gondii* is independent of microtubules, whereas during mitosis the microtubules are required for anchoring the nucleus to the centrosome (Farrell and Gubbels, 2014). The spindle microtubules emanate from the centrosome, which always remains in close proximity to the centrocone (Dubremetz and Elsner, 1979; Morrissette and Sibley, 2002). Notably, the dynamics of spindle microtubule assembly are peculiar. In *T. gondii* tachyzoites, the centrosome resides on the apical side of the nucleus during G1-phase, but prior to its division the centrosome migrates to the basal side of the nucleus (Hartmann et al., 2006). It is on the basal side of the nucleus, to which the centrocone co-migrates, that the spindle microtubules start assembling. However, before completing mitosis, the centrocone and centrosome rotate back to the apical side of the nucleus where they associate with the apicoplast and Golgi apparatus to ascertain their correct partitioning (Chen et al., 2015b). Why this rotation happens, whether the whole nucleus rotates, and whether this also happens when the D&S rounds are not connected to the budding cycle are unknown.

The centromere connects to the (+)-end of the spindle microtubules of the kinetochore, of which the Nuf2/Ndc80 complex and degenerate SPC24 and SPC25 proteins have been described in *T. gondii* and *Plasmodium* (Farrell and Gubbels, 2014; Zeeshan et al., 2020b). In addition, the chromosomal passenger complex containing inner centromere protein (INCENP) and Aurora kinase 1 (Ark1) identified in *T. gondii* dynamically associates with the centromeres and is critical for completion of mitosis (Berry et al., 2018). However, none of the other typical kinetochore proteins described for other eukaryotes are present in the apicomplexan genomes, and proteomic approaches have to date not identified additional kinetochore components. This suggests the kinetochore is either very reduced or unusual. Ultrastructural observations on *T. gondii* support at maximum one, but possibly less than one microtubule per kinetochore (Swedlow et al., 2002). This unusual configuration fits with the observation of clustered centromeres, which would also not require capturing each individual chromosome by the spindle microtubules. The slow second part of the bimodal S-phase may represent the timing of this process, although this period has also been suggested as a distinct pre-mitotic checkpoint that replaces the typical G2-phase seen in higher eukaryotes (White and Suvorova, 2018), or, alternatively, it could represent the timing of centrosome duplication associated with nuclear rotation.

The next step is the separation of the chromosome sets. Kinesin motors have been characterized in *Plasmodium berghei*: kinesin-5 (Eg5 ortholog) and kinesin-8X associate with the spindle across various developmental stages, including schizogony, but surprisingly, were only essential during the mosquito stages (Zeeshan et al., 2019; Zeeshan et al., 2020a). Phenomenologically, completion of mitosis is not necessarily concluded by karyokinesis, which is skipped in some apicomplexan division modes. A big question germane to the division modes where D&S is not followed by karyokinesis is the licensing of DNA replication: how is the parasite able to re-enter S-phase multiple times per nucleus, while making sure that each time only a single copy of each set of chromosomes is made? Licensing has only been studied in *P. falciparum* schizogony and interestingly, a connection has been made with a cyclin dependent kinase (Cdk), which might be a mechanism to control this (Deshmukh et al., 2016). Another observation here is that progression through multiple rounds of D&S is synchronous in the polyploid nucleus of *Sarcocystis neurona* undergoing endopolygeny (Vaishnava et al., 2005), which contrasts with the asynchronous D&S plus karyokinesis rounds for each nucleus observed in schizogony and endopolygeny with karyokinesis as seen during *T. gondii* and *Cystoisospora suis* merogony (Gubbels et al., 2020). At least there is a mechanism in place to keep complete sets of chromosomes organized: through their centromere clustering and association with the nuclear envelope and centrosome (Vaishnava et al., 2005).

### Karyokinesis

Karyokinesis is optional after each round of D&S, but clearly it is required to endow each daughter zoite with a complete set of chromosomes. Only little information regarding the machinery and the mechanism are available. In the division modes producing two daughters per division round, it appears as if the basal complex at the base of the IMC scaffold drives nuclear fission, as the nucleus is stretched out between the two forming daughters (**Figures 1** and **2A, B**). However, in multi-nuclear schizonts across division modes, karyokinesis completes without the need for the IMC scaffold, so the mechanism must be distinct. Ultrastructural studies have not revealed any electron dense rings that could indicate a contractile ring driving fission of the nuclear envelope (Dubremetz, 1973; Dubremetz and Elsner, 1979; Francia et al., 2020; Rudlaff et al., 2020). There is no information regarding the cytoskeleton of the nucleus as typical nuclear lamins are not found in the genome. The nuclear envelope harbors typical FG-proteins assembled into the NPC (Weiner et al., 2011; Bandini et al., 2016; Courjol et al., 2017; Kehrer et al., 2018), which themselves are unlikely to have a function in karyokinesis. Proteomics studies of the *Plasmodium* nucleus (Oehring et al., 2012) and *Toxoplasma* whole tachyzoite proteomics (Barylyuk et al., 2020) harbor many candidates for this process, but they have thus far not been mapped.

A recent study of the *P. falciparum* Mini-Chromosome Maintenance Complex Binding Protein (PfMCMBP) reported that this protein is involved in coordinating chromosome seggregation and karyokinesis (Absalon and Dvorin, 2020). PfCMBP depletion resulted in spindle microtubules connecting multiple nuclei, while impacting the appearance of the centrosome. Since cytokinesis progressed normally, PfCMBP likely acts on the centrocone or the inner-core of the centrosome, but whether the defects in the spindle are a primary or secondary defect could not be clearly differentiated (Absalon and Dvorin, 2020). Another factor recently shown to affect mitotic and karyokinesis progression during *Plasmodium* schizogony is protein phosphatase 1 (PP1). In general, PP1 functions in mitotic exit and cytokinesis. PP1 depletion during *Plasmodium* schizogony resulted in reduced DNA replication and reduced nuclear centers, which suggested a defect in karyokinesis (Paul et al., 2020). Again, it is not completely clear if the karyokinesis defect is primary or secondary. Lastly, an actin related protein, ARP4, localizing to the nucleus of *T. gondii* tachyzoites, is critical for chromosome segregation and/or partitioning, but the mechanism remains uncharacterized (Suvorova et al., 2012). In summary, our knowledge of karyokinesis is characterized by many more unknowns than knowns.

### Zoite Assembly Through Daughter Budding

The overarching principles of asexual zoite budding across stages are: 1. bud nucleation on the centrosome; 2. parallel assembly of all three key components (microtubules, alveoli, IMC meshwork) in an apical to basal direction; 3. anchoring of inherited organelles to the division machinery; 4. *de novo* assembly of secretory organelles; 5. a contractile basal complex (BC) that tapers the zoites on the basal end (Anderson-White et al., 2012; Francia and Striepen, 2014).

**Figure 3:**
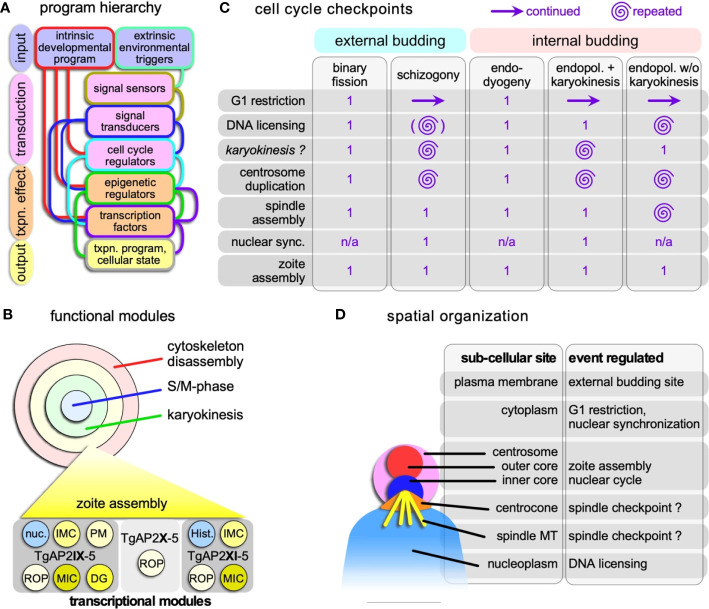
Hierarchical and spatial organization of the division modes. **(A)** Schematic general representation of how the circuitry underlying the various division modes is organized. The lines connecting the boxes represent the general paths on how the various steps direct each other, although there are likely additional feedback loops between the various levels not represented in the schematic. The color of the connecting lines indicates the directionality: the color from the outline of the box from which they originate is the starting point, and directs the box of different color to which they connect. **(B)** Schematic representation of the four functional modules that can be strung together in various ways into the various apicomplexan division modes. Each of these modules is composed of a collection of transcriptional programs controlled by (combinations) of transcription factors. As a hypothetical example of how functional module is composed of a collection of transcriptional modules, the transcriptional collection making up the zoite assembly (daughter budding) module is shown at the bottom. An example of two key transcription factors with proven roles in the *T. gondii* budding cycle are shown at the bottom (Walker et al., 2013; Wang et al., 2020a; Khelifa et al., 2021); TgAP2X-5 cooperatively recruits TgAP2XI-5 to rhoptry gene promoters (Lesage et al., 2018a). Note some overlap with the nuclear cycle in the form of histones (Hist.) and other nuclear factors (nuc.). ROP, rhoptry proteins; MIC, microneme proteins; DG, dense granule proteins. **(C)** Cell cycle checkpoints in Apicomplexa and how they are organized and repeated multiple times in the various division modes. Zoite assembly is only executed once in each cycle. Continued G1 reflects the state of the cytoplasm, while the nucleus goes through multiple and repeated cycles; the G1 restriction point has to be passed to permit that. There is currently no experimental support that karyokinesis is a true cell cycle checkpoint as checkpoints were defined in *T. gondii* tachyzoites (Suvorova et al., 2015; Alvarez and Suvorova, 2017; Naumov et al., 2017). The brackets around DNA licensing in schizogony indicate this is optional within the same nucleus, as limited karyokinesis is observed. Nuclear sync. refers to the arrest of asynchronously replication nuclei in interphase before they all synchronously continue into a final nuclear cycle connected to zoite assembly. n/a: not applicable. **(D)** Schematic representation of the subcellular sites in the apicomplexan cell where the various controls reside. Assignments regarding the centrocone and centrosome are largely derived from work on *T. gondii* tachyzoites (Suvorova et al., 2015; Alvarez and Suvorova, 2017; Naumov et al., 2017) and has as yet only been limitedly validated for the external division modes. The site of the spindle checkpoint is in the centrocone, through which the microtubules enter the nucleoplasm. Whether the checkpoint resides on the microtubules or in the centrocone is unclear.

The ‘bipartite centrosome’ model proposed for *T. gondii* tachyzoite formation is likely a universal model that connects a final round of the nuclear cycle, comprising the D&S as well as karyokinesis, with the budding cycle (Suvorova et al., 2015): the inner-core of the centrosome coordinates the nuclear cycle, whereas the outer-core of the centrosome coordinates the budding cycle (**Figure 3D**). The inner-core can proceed without activation of the outer-core to generate polyploid schizonts, but activation of the outer-core requires simultaneous activation of the inner-core (Suvorova et al., 2015). The appearance of the centrosome differs somewhat between parasite species, for example, *Plasmodium* asexual parasites do not have centrioles (Mahajan et al., 2008), whereas the *T. gondii* centrosome carries two parallel centrioles (Francia et al., 2015; Morlon-Guyot et al., 2017) (**Figures 2I, J**). Despite these difference in presentation, mechanistically, the role of the centrosome in initiating zoite assembly is universally conserved. The *T. gondii* centrosome is anchored to the nucleus by the spindle microtubules on one side (Farrell and Gubbels, 2014) and in the apical end of the daughter bud by a striated rootlet fiber protein assembly (SFA) (Francia et al., 2012).

The sequential assembly of the cortical cytoskeleton scaffolds of the daughter buds has been studied in most detail in the *T. gondii* tachyzoites, which we use here as a primary guide for the other species (Anderson-White et al., 2012; Francia and Striepen, 2014; Kono et al., 2016; Gubbels and Morrissette, 2020). The onset of budding is mediated by an F-box protein localizing to the centrosome, TgFBXO1, which likely triggers a switch in budding competence of the outer-core (Baptista et al., 2019). The sub-pellicular microtubules and the IMC are assembled in concert and deposited in an apical to basal direction. The apical polar ring serves as a microtubule organizing center for the sub-pellicular microtubules whereas the alveolar membranes are delivered through the secretory pathway by the alveolate specific Rab11B GTPase (Agop-Nersesian et al., 2010). Palmitoylation of IMC proteins anchors them into the alveolar vesicles, which is an essential step across species and division modes (Dogga and Frenal, 2020; Wang et al., 2020b). There is a wide variety of proteins localizing to the IMC, but proteins with an alveolin repeat are intermediate filament-like and assemble in a meshwork of proteins undergirding the alveolar vesicles (Gould et al., 2008; Anderson-White et al., 2011; Kono et al., 2012; Tremp et al., 2014; Chen et al., 2015a; Goodenough et al., 2018). The IMC soluble proteins (ISP), which do not become crosslinked in the meshwork, are critical in both *T. gondii* tachyzoite and *Plasmodium* ookinete formation (Beck et al., 2010; Fung et al., 2012; Wang et al., 2020b). Finally, maturation steps are needed to consolidate the most apical structures in *T. gondii* (Back et al., 2020; Tosetti et al., 2020) as well as the proteolytic processing of alveolin protein TgIMC1 (Mann et al., 2002).

Regarding the inherited single copy organelles, the Golgi apparatus and the apicoplast associate with the centrosome to facilitate their partitioning in each daughter (Striepen et al., 2000; Pelletier et al., 2002). The mechanism of mitochondrion partitioning is pluriform: in *T. gondii* the mitochondrion enters the daughter buds last and is anchored to the IMC scaffold (Jacobs et al., 2020), whereas in *Plasmodium* it hitchhikes by anchoring to the apicoplast (Van Dooren et al., 2005; Stanway et al., 2011).

Non-inherited secretory organelles like the micronemes, rhoptries and dense granules are largely assembled *de novo* through the secretory pathway when the daughter cytoskeleton is growing (Nishi et al., 2008; Periz et al., 2019). The micronemes and rhoptries (Beck et al., 2013) are anchored directly to the daughter scaffold whereas the scattered nature of dense granule distribution suggests a stochastic partitioning mechanism.

The leading edge of the forming daughter cell known as the basal complex (BC; **Figure 2B**) is defined by the presence of MORN1 (Ferguson et al., 2008; Kono et al., 2016). The BC keeps the daughter bud together as its absence results in fraying microtubules and prevents completion of *T. gondii* daughter budding (Gubbels et al., 2006; Heaslip et al., 2010; Lorestani et al., 2010). In both *T. gondii* endodyogeny (Frenal et al., 2017) and *Plasmodium* schizogony (Rudlaff et al., 2019) the BC constricts to taper the parasites toward the basal end. Although sometimes a cytoplasmic bridge remains between the emerging daughter zoites, this last step completes the budding process.

### Cytoskeleton Disassembly

The timing of mother cytoskeleton disassembly has a tremendous impact on the orchestration of cell division. In the external division modes this is the first step, with relatively simple logistics compared to internal budding where this is the final step and thereby coincides with the assembly of new daughters. We will first review the phenomenology in these variations and then discuss insights in putative mechanisms underlying differential mother and daughter cytoskeleton stability in the internal budding modes.

Cytoskeleton disassembly in external budding cell division modes has been studied for both the liver form and the red blood form schizogony cycles of *Plasmodium* as well as for *Babesia* binary fission. Upon entry of *Plasmodium* sporozoites in the liver cell, regular spaced breaks start forming in the IMC in the center around the nucleus and the cytoplasm starts to bulge out (**Figures 2C, D**) (Verhave and Meis, 1984; Jayabalasingham et al., 2010). The IMC detached from the plasma membrane accumulates in the cytoplasm, while the two distal ends of the sporozoite gradually retract and disappear over an approximately 24 hrs while the zoite gradually rounds up (**Figure 2D**) (Meis et al., 1985; Kaiser et al., 2003; Jayabalasingham et al., 2010). During this process, the alveolar cytoskeleton is reorganized into dense lamellar arrays within the cytoplasm and is partially expulsed in bloc by converting parasites (Jayabalasingham et al., 2010). At the same time the rhoptries and micronemes are degraded. Micronemes have been shown to compartmentalize into large exocytic vesicles which are discharged into the vacuolar space (Jayabalasingham et al., 2010). Clearance of the micronemes is mediated by ATG8 carrying vesicles, which suggests the mechanism involves the autophagy pathway (Voss et al., 2016). The fate of the microtubule skeleton has not been exhaustively researched but at least initially they remain associated with the IMC while it is breaking up (Verhave and Meis, 1984). Surprisingly, insights on merozoite cytoskeleton disassembly in the red blood cell are very sparse, which in part could be due to the speed of this process: 15 min after completing invasion only small IMC membrane pieces remain (Riglar et al., 2013). Upon entry of *Babesia bigemina* merozoites into the red blood cell a highly symmetric disassembly of the IMC was observed (**Figure 2E**) (Gubbels et al., 2020). The IMC breaks up consistently in 5-6 regularly sized fragments, while the cell is rounding up. Although not all IMC fragments are completely cleared up before zoite assembly starts, the majority of IMC fragments turns over swiftly. How and where the site of the breaks in the IMC are generated is unknown.

Insights on mother cytoskeleton disassembly during internal budding are derived from *T. gondii* endodyogeny and *S. neurona* endopolygeny. During endodyogeny the mother’s IMC disassembles and retracts in a very organized, apical to basal direction while at the same plasma membrane is deposited on the emerging daughter parasites giving the appearance of a ‘zippering’ mechanism (Morrissette and Sibley, 2002; Anderson-White et al., 2012) (**Figures 2B, F–H**). Many IMC proteins are present in the ubiquitome suggesting degradation of mother components by the proteasome (Silmon De Monerri et al., 2015), although a fraction of the IMC proteins is recycled in the emerging daughters (Ouologuem and Roos, 2014). On the other hand, the mother’s conoid and the subpellicular microtubule cytoskeleton remain largely intact and slowly migrate basally into the residual body where these components are further degraded (Morrissette and Sibley, 2002) (**Figures 2I, J**). The residual body forms at the basal ends of the emerging daughters and is considered a digestive compartment to recycle the disposed remnants of the mother. It typically rapidly shrinks in size following the completion of division and is enriched in ubiquitinated proteins (Dhara and Sinai, 2016). Besides, expulsion, proteasomal degradation and recycling, autophagy is another possible mechanism of organelle turnover but there is currently no experimental support for the latter. The disappearance of the mother’s micronemes and rhoptries largely coincides with the onset of cytoskeleton disassembly during endopolygeny. In the case of *S. neurona* endopolygeny, the micronemes disappear halfway during the division cycle (Vaishnava et al., 2005), which is well-ahead of cytoskeleton disassembly occurring just prior to daughter emergence (Dubey et al., 2017). Moreover, the timing of cytoskeleton disassembly during *T. gondii* and *Cystoisospora suis* endopolygeny also overlaps with daughter emergence (Dubey et al., 2017; Gubbels et al., 2020) (**Figure 2K**). Thus, a variety of processes, some more conserved than others and with different timing, are employed for organelle turnover and cytoskeleton disassembly across division modes.

Internal budding provides an additional challenge: balancing the simultaneous maturation of the daughter cytoskeletons with the disassembly of the mother. Firstly, maturation requires proteolytic processing of IMC1, which triggers a transition to a detergent resistant conformation of the alveolar protein meshwork (Mann et al., 2002). Secondly, GAP45 is a dually acylated protein that spans the space between the IMC outer membrane and the plasma membrane (Frenal et al., 2014) and is deposited in apical to basal direction on the emerging daughter which tracks closely with removal from the retracting mother’s IMC (**Figures 2B, G, H**) (Gaskins et al., 2004; Gilk et al., 2009). It is important to note that GAP45 is deposited at the time of budding during the externally bound assembly of *Plasmodium* merozoites (Rees-Channer et al., 2006), which could indicate that the differential stability is a relatively simple function of hooking up the IMC to the plasma membrane. Thirdly, there is a variety of IMC resident proteins that localize differentially to mother and daughter cytoskeletons [for details, see (Gubbels et al., 2020)]. Examples of such differential localization in *T. gondii* are provided in **Figures 2F, K**, where IMC3 predominantly stains the budding daughters and IMC7 marks the mother’s cytoskeleton (Anderson-White et al., 2011; Dubey et al., 2017). These may contribute to differential stability, but to date no single factor has provided a satisfactory answer, which could indicate redundant mechanisms controlling this very critical step.

## When, Where, and How Decisions Are Made and Executed

### Commitment to Division or Differentiation

The first choice faced by the parasite at the start of the replication cycle is whether to divide by internal versus external budding. This is a not truly a choice since it is a genetically fixed commitment across all asexual division modes for a particular parasite species, and the switch between developmental stages is with few exceptions, unidirectional. Transmission through the life-cycle with various developmental forms is the key to apicomplexan survival. During some stages, parasites will go through multiple rounds of asexual proliferation, or meiosis for sexual recombination. For instance, *Plasmodium* sporozoites invade liver cells, within which they undergo one round of schizogony to produce tens of thousands of merozoites within a single cell, that then are released into the circulation to invade red blood cells. Once in red blood cells, parasites can cycle through multiple and continuous asexual erythrocytic cycles, or at a low frequency can switch from asexual replication into a sexual differentiation pathway to form gametocytes. Frequency of switching to sexual differentiation can be in response to external triggers (Duraisingh and Skillman, 2018). In essence, it may appear that liver-stage parasites cannot vary within their development stage, but blood-stage parasites do. We note that there are exceptions such as *Plasmodium vivax* sporozoites that following the invasion of liver cells form either hepatic schizonts or quiescent hypnozoites. This indicates that reprogramming is a possible feature within all developmental stages. Current insights define the developmental stage by environmentally informed epigenetic reprogramming of the genome in combination with transcription factors that fine tune the timely expression of genes throughout the division cycle (Bunnik et al., 2018; Fraschka et al., 2018; Hoeijmakers et al., 2019; Toenhake and Bartfai, 2019; Hollin and Le Roch, 2020; Waldman et al., 2020; Hollin et al., 2021) (**Figure 3A**). Typically, these reprogramming steps are initiated in the division cycle preceding differentiation (Radke et al., 2003; Brancucci et al., 2017; Sinai and Suvorova, 2020; Venugopal et al., 2020).

Chromosome organization and chromatin are involved at several levels: 1. the three-dimensional organization of chromosome in the nucleus; 2. the organization is euchromatin vs. heterochromatin; 3. local chromosome accessibility at the promoter by post-translational histone tail modifications (recently reviewed by (Hollin and Le Roch, 2020)). 3D chromosome organization is in general regulated by long non-coding RNA (lncRNA) molecules. Although non-coding RNA in Apicomplexa has not been extensively studied (Li et al., 2020), there is one validated example of this mechanism in Apicomplexa: regulation of *P. falciparum* gametocytogenesis is controlled by an antisense lncRNA transcribed from the *gdv1* locus, which represses GDV1 (Filarsky et al., 2018). The role of GDV1 is to evict PfHP1 from histone 3 K9me3 sites to turn on gene expression and thereby committing to gametocytogenesis (Filarsky et al., 2018; Rea et al., 2018). This immediately highlights the role of histone modification. Over 240 different post translational modification have been detected on *Plasmodium* histones, of which only a small fraction is functionally understood (Saraf et al., 2016). Genome-wide studies in *P. falciparum* and *T. gondii* have revealed that active promotor regions are marked by a complex pattern of histone H3 and H4 methylation and acetylation (Gissot et al., 2007; Gupta et al., 2013; Gupta and Bozdech, 2017; Fraschka et al., 2018; Toenhake and Bartfai, 2019).

Histone modifications are deposited by epigenetic writer enzymes, whereas readers relay this information into a transcriptional response. The epigenetic machineries have recently been reviewed for *Plasmodium* (Duraisingh and Skillman, 2018) and *T. gondii* (Kim, 2018) and comprise approximately 3-10 member families of histone deacetylases (HDACs), putative histone acetyltransferases, methyltransferases, and demethylases. These enzymes (are predicted to) add and remove methyl and acetyl groups to different lysines found in histone tails. However, enzymatic family expansion can be tailored to specific needs. For example, *T. gondii* harbors 20 lysine methyltransferases, of which some act on histones, but others have validated roles in other processes such as engaging the invasion machinery (Heaslip et al., 2011) or the apical annuli (Engelberg et al., 2020). Another example is the allelic exclusion for antigenic variation elaborated in *P. falciparum*, which is controlled by histone modification (this mechanism is absent from *T. gondii*). In summary, specialized roles and requirements for acetylation and methylation enzymes in each system is reflected in their writer and eraser repertoires, but the machinery dedicated to regulation of the division modules is likely quite conserved.

The repertoire of epigenetic readers contains 7 bromodomain in *P. falciparum* (Hoeijmakers et al., 2019), which with 12 members is almost twice as large in *T. gondii* (Jeffers et al., 2017). It is not clear whether this is due to lineage specific functions as only two members (two distinct GCN5 factors) have been characterized experimentally in *T. gondii* (Wang et al., 2014; Harris et al., 2019), or are simply a scale function of the 2.5-fold larger *T. gondii* genome relative to *P. falciparum* (Iyer et al., 2008). Particular bromodomain proteins can have very specific roles e.g. *P. falciparum* PfBDP1 binds to chromatin at transcriptional start sites of invasion-related genes and directly controls their expression (Josling et al., 2015). Other reader proteins comprise small groups of PHD domain and chromo domain proteins (Gissot et al., 2012; Wang et al., 2014; Harris et al., 2019; Hoeijmakers et al., 2019). Overall, it appears that these machineries are very comparable between *T. gondii* and *Plasmodium* spp. while examples support specialization to some lineage specific functions.

Overall, chromatin openings within 2 kb upstream of the transcribed genes correspond very well with the temporal/developmental changes in transcriptional levels observed throughout the *P. falciparum* schizogony cycle (Toenhake et al., 2018). Variation in histone modifications correlate with timing of expression during the *Plasmodium* schizogonic replication cycle (Gupta et al., 2013), suggesting a tightly orchestrated writer/reader machineries. The identification of bromodomain proteins regulating specific gene sets e.g. in the *P. falciparum* invasion genes (Josling et al., 2015) in combination with the PfAP2-I transcription factor (Santos et al., 2017) seems to be a larger shared principle as additional combinations of epigenetic readers in concert with ApiAP2 and other transcription factors have been reported (Filarsky et al., 2018; Harris et al., 2019; Hoeijmakers et al., 2019; Farhat et al., 2020). Altogether, these findings point at integrated epigenetic and transcriptional machineries that are finely tuned to regulate specific division modules.

### Cell Cycle Progression

Where genome accessibility sets the stage for the cell division mode, progress through the cell division process itself is governed by oscillating mRNA levels resulting in continuous wave-like patterns of gene expression. This has been elegantly shown using microarrays for the *P. falciparum* schizogenic (Bozdech et al., 2003; Le Roch et al., 2003) and *T. gondii* endodyogenic cycles (Behnke et al., 2010). Recently, these results have been confirmed by single cell RNA sequencing (scRNA-seq) data in both systems, which also led to the identification of overlaps in the transcriptional networks underlying cell division modules (Howick et al., 2019; Xue et al., 2020). Checkpoints in cell division cycle progression are governed by the general eukaryotic cyclins and cyclin dependent kinases (Cdk) class of regulators. However, the wiring is quite divergent in the Apicomplexa as protein levels of cyclins are often not going up and down through the cell cycle, and a notably distinct, cyclin-independent Cdk-related kinase (Crk) family that does not pair with cyclins is critical [for recent reviews see (Matthews et al., 2018; White and Suvorova, 2018)]. Overall the Cdks and Crks are relatively well conserved, with 7 representatives in both *P. falciparum* and *Theileria annulata* and, 10 members in *Toxoplasma* (Alvarez and Suvorova, 2017).

#### The Cell Cycle ApiAP2 Connection

The general eukaryotic mechanism is that Cdks relay their activation state by phosphorylation of transcription factors. The typical transcription factors seen in mammals, such as E2F and RB, are absent from most apicomplexan genomes (Oberstaller et al., 2014; White and Suvorova, 2018). Therefore, it has been suggested that the expansive ApiAP2 family of transcription factors has replaced the need for these factors. The ApiAP2 transcription factors are related to the plant family Apitella2 (AP2) factor, which typically are defined by one, but can contain up to three, AP2 DNA binding domains as the only recognizable domains in >100 kDa proteins that otherwise are largely unstructured (Balaji et al., 2005; De Silva et al., 2008; Painter et al., 2011; Jeninga et al., 2019). Surveys of the ApiAP2 transcription factor repertoire across the Apicomplexa and their free-living Chromerid relatives have identified very few generally conserved factors (Woo et al., 2015). Germane to the Coccidia and Hemosporidia parasite group considered here, the gamut ranges from the low 20s in *Babesia* spp. to 64 ApiAp2 encoding genes in *T. gondii* (Reid et al., 2014; Jeninga et al., 2019). More importantly, these studies highlight a dramatic expansion of the ApiAP2 repertoire in the Coccidia and Hemosporidia relative to the sister group of *Cryptosporidium* spp. (outside the Coccidia-Hemosporidia group). Coccidia have a genome size 30% larger than the *Babesia* spp. but only encode ~18 ApiAP2 proteins, which is less than the 22 encoded by *Babesia* spp (Oberstaller et al., 2014). The relatively small number of ApiAP2s in *Cryptosporidium* spp. is compensated by the presence of E2F family of transcription factors typically regulated by Cdks/Crks. Such E2F factors typically regulated by Cdks/Crks are absent from the Coccidia and Hemosporidia, the model is that the unconventional yet fairly conserved cyclin/Cdk/Crk network in this group acts on the diversified spectrum of ApiAP2 factors (Oberstaller et al., 2014; White and Suvorova, 2018).

An additional key insight is that the number of ApiAP2 factors correlates with genome size across the Hemosporidia and Coccidia, and not with the complexity of their life cycles (Oberstaller et al., 2014; Reid et al., 2014; Blazejewski et al., 2015; Woo et al., 2015; Alzan et al., 2016). This is consistent with a general genome scaling law correlation even seen in bacterial systems (Van Nimwegen, 2003). Since the number of ApiAP2 factors is very variable and their sequence conservation poor, the individual wiring in each parasite seems to be its own unique tapestry. In other words, the mechanism of regulation is conserved (cyclin/Cdk/Crk), but the players (ApiAP2s) are highly diverse. This is most likely the key defining factor in the organization of the various cell division modes in this group of parasites (**Figure 3A**).

As an example of one of the better understood transcriptional programs consider the zoite assembly module of *T. gondii* endodyogeny (**Figure 3B**). Three ApiAP2 factors are largely responsible for forming new daughter buds: in a TgAP2X-5 fashion, TgAP2XI-5 drives many secretory organelle proteins (Walker et al., 2013; Lesage et al., 2018b) whereas TgAP2IX-5 controls the expression of most cytoskeleton scaffold genes next to additional secreted proteins (Wang et al., 2020a; Khelifa et al., 2021) (**Figure 3B**). Repression of TgAP2IX-5 actually results in schizonts with multiple nuclei reminiscent of a switch to endopolygeny (Khelifa et al., 2021). Releasing the schizont stage by turning TgAP2IX-5 back on even reinitiates zoite assembly, and albeit at a low rate, resulting in complete budding of viable parasites that subsequently divide by endodyogeny. Merozoites forming and assembling in the *Plasmodium* erythrocytic cycle show a similar transcriptional program of coordinated expression of distinct groups of invasion factors (Hu et al., 2010), which at least requires the PfAP2-I transcription factor (Santos et al., 2017), as well as the bromodomain protein PfBDP1 (Josling et al., 2015). In addition, scRNA-seq data across developmental stages and division states of *Plasmodium* spp. (Howick et al., 2019; Real et al., 2020) and *T. gondii* (Xue et al., 2020) consolidated the existence of specific developmental programs and advanced the insights into the exact transcriptional states of individual cells. Indeed, comparison of *Plasmodium* and *T. gondii* data sets of schizogony and endodyogeny, respectively, revealed concerted expression of mitochondrial, centrosome, DNA replication, IMC, and microtubule, gene sets despite the variations in ApiAP2 repertoires (Xue et al., 2020). Altogether, these observations support a model of combinations of species- and stage-specific transcription factor combinations as outlined in **Figure 3C** that define the functional modules supporting each stage of the division cycle.

#### Spatiotemporal Organization of Cell Cycle Checkpoints

Mitosis in eukaryotes typically requires a spindle assembly checkpoint (SAC) to prevent mitotic progression if not all chromosomes are attached to spindle microtubules. Since the apicomplexan chromosomes remain largely clustered throughout the division cycle, this seems to be less critical, or at least might be organized differently. It is therefore not completely unsurprising that most of the SAC components are absent from the Apicomplexa (Kops et al., 2020). However, the typical molecules in chromatid cohesion and separation (e.g. cohesin, separase) and at least some members of the anaphase promoting complex (APC) are conserved in the genomes (Eme et al., 2011). And as mentioned before, there is an indication that Cdks are involved in mitotic progression of *P. falciparum* (Deshmukh et al., 2016). The systematic dissection of *T. gondii* cyclins, Cdks and Crks identified 5 checkpoints in the endodyogeny cycle: 1. G1 restriction; 2; DNA licensing 3. centrosome duplication; 4. spindle assembly; 5. zoite assembly (Alvarez and Suvorova, 2017; Naumov et al., 2017) (**Figure 3C**). Interestingly, ‘spindle assembly’ during mitosis is a checkpoint mediated by TgCrk6.

The dissection of cell cycle factors highlighted unique aspects of the apicomplexan division cycles [reviewed in (Matthews et al., 2018; White and Suvorova, 2018)]. In particular during the multi-daughter schizogony and endopolygeny strategies, the parasite passes through several of these checkpoints multiple times. The distinct spatial organization of these checkpoints is the feature that permits such check point uncoupling from the general progression of passing each point only once (**Figure 3D**). The bipartite centrosome is an import platform in the spatial uncoupling since the multiple nuclear replication cycles are controlled by the inner-cores and execution of zoite formation is coordinated by the outer-core (Suvorova et al., 2015; White and Suvorova, 2018). During schizogony and endopolygeny with karyokinesis the D&S+karyokinesis cycles for each nucleus become unsynchronized. The current model in Apicomplexa is that the centrosome cycles are uncoupled between individual nuclei (Gerald et al., 2011; Roques et al., 2015; Gubbels et al., 2020). The mechanistic basis of this resides in the state of maturity of the centrosomes following division: the mother centrosome is mature already whereas the daughter requires more time to mature. Consequently, the mother centrosome is sooner primed for another round of division. Centrosome maturation in well-studied systems is based on recruiting proteins to the peri-centrosomal matrix (PCM), but so far no robust PCM candidates have materialized in the Apicomplexa (Chen and Gubbels, 2019).

However, the centrosome maturation model does not explain the division modes that skip karyokinesis after each round of D&S, since in this case all mitotic spindles in the same nucleoplasm are tightly synchronized (Vaishnava et al., 2005; Gubbels et al., 2020). Skipping karyokinesis after each D&S round can either be strictly organized as seen in the single large polyploid nucleus during *S. neurona* endopolygeny or it can be a more stochastic event as seen during sporogony of *Plasmodium* spp. where only occasionally nuclei skip karyokinesis to form lobed nuclei (Gubbels et al., 2020). A factor diffusing through the nucleoplasm to keep the cycles synchronized is the most likely scenario. Factors satisfying this bill are the mitotic regulators TgCrk5 and ‘essential for chromosome replication 1 (ECR1), which in *T. gondii* localize to the spindle pole during mitosis, re-distribute to the nucleoplasm upon completing mitosis while they are degraded by a ubiquitination-based mechanism during interphase (Naumov et al., 2017; White and Suvorova, 2018). However, the *P. falciparum* ortholog PfCrk5 localizes permanently in a speckled nuclear pattern, regardless of the stage of the nuclear or cell cycle (Dorin-Semblat et al., 2013). Recent work on PbCrk5 revealed its phosphorylation targets are the DNA replication licensing machinery and is in a complex with cyclin SOC2 during gametogony and sporogony (Balestra et al., 2020). However, SOC2 is not conserved in *T. gondii*, suggesting a divergent wiring consistent with its distinct localization pattern. It is therefore possible these controls are a distinctive feature between internal and external budding though they might share a similar function in licensing DNA replication and mitotic progression. Overall, the controls and mechanism of karyokinesis are still largely undefined. Even whether karyokinesis is truly a checkpoint is debatable, as it is fairly optional in nature if the zoite assembly mode is not simultaneously activated. It might therefore also be hardwired together with the zoite assembly module.

There is another checkpoint during acting on repetitive nuclear cycles that is mediated by PfCrk4 in *P. falciparum* schizogony (Alvarez and Suvorova, 2017; Ganter et al., 2017; White and Suvorova, 2018). PfCrk4 localizes to the nucleoplasm, is required for DNA replication during schizogony and is involved in activating the origin of replication machinery. Although blocking PfCrk4 leads to an early arrest in schizogony resulting is reduced DNA replication. Depletion of the ortholog TgCrk6 during *T. gondii* does not prevent the onset of budding in *Toxoplasma* endodyogeny, yet mitosis is arrested and behaves like a spindle checkpoint. Hence, the orthologous proteins in *T. gondii* and *P. falciparum* appear to control different checkpoints, but a definitive answer will require its experimental assessment in an endopolygeny system wherein multiple nuclei are present.

#### Genetic Programs and Environmental Input Drive the Number of Nuclear Cycles

The number of offspring per apicomplexan division round varies from two (endodyogeny and binary fission) to several orders of magnitude higher (10,000s in schizogony). At the furthest extreme are several bovine-infecting *Theileria* spp. of which the schizonts in the white blood cells trigger transformation of their lymphocyte host cells (i.e. leukemia) resulting in limitless division and expansion of the parasite’s schizont stage along with their host cell (Luder et al., 2009; Chakraborty et al., 2017). A less pronounced manifestation of host cell manipulation is the *Plasmodium* induced liver cell expansion permitting the production of ~90,000 merozoites from a single sporozoite infection (Vaughan and Kappe, 2017). The other outer limit is found in the closely related large *Babesia* species which have lost all capacity to make more than two daughters per division round, regardless of their developmental stage (Mehlhorn and Shein, 1984; Gubbels et al., 2020). Thus, some parasites have the capacity to modify their environment to accommodate their number of offspring as long as the host cell can be manipulated. Since red blood cells cannot be manipulated to expand, it sets a physical limit on the number of progeny that can be accommodated.

A differently flavored example of response to environmental cues is the quorum sensing seen in *Toxoplasma* endodyogeny. During the intracellular replication cycle the parasite progressively accumulates phosphatidic acid (PA) in the vacuolar space while at the same time the pH drops (Roiko et al., 2014; Bisio et al., 2019). When these variables reach certain thresholds, the parasite responds by egressing, which blocks further initiation of parasite replication [egressing parasites already in division will however complete their division in the extracellular milieu (Gaji et al., 2011)] (Bullen et al., 2019). However, if there are disruptions in any step or leg of the signaling pathway or even execution steps, the parasite remains in the default division mode and generates much larger vacuoles than typically observed (Chandramohanadas et al., 2009; Kafsack et al., 2009; Farrell et al., 2012).

Extending on the above observation, aspects of lipid metabolism appear to be common signals triggering stage transitions across the Apicomplexa. For instance, phospho-inositol (PIP) metabolism activates PKG toward egress in *Plasmodium* (Brochet et al., 2014), whereas linoleic acid is critical to activate the sexual cycle in *T. gondii* (Martorelli Di Genova et al., 2019). *T. gondii* sexual development initiates with merogony, which unfolds by division by endopolygeny with karyokinesis. Interestingly, multi-daughter budding *T. gondii* of four tachyzoites per division rounds can be induced by modulating the availability of lipids (Lige et al., 2011), membrane through Golgi-mediated trafficking (Stedman et al., 2003), or IMC cytoskeleton components (Beck et al., 2010; Dubey et al., 2017). It therefore appears that timely availability of lipids and/or membrane is critical for the daughter bud initiation function of the outer centrosome core. Overall, the parasite needs to balance efficient use of resources against the timely escape of immune surveillance and the ability to still form mature zoites.

Considering the phenomenon of endomitosis, the Apicomplexa stand out by their accuracy and efficiency of reducing polyploid cells into cells of reduced, normal ploidy (Lee et al., 2009). The most striking aspect of this process is that asynchronously dividing nuclei arrest in interphase and then undergo a synchronized final round of D&S+karyokinesis linked to budding to produce an even number of daughter zoites. There are two signaling events: 1. prevent re-entry into S-phase until all nuclei are in interphase; 2: re-enter S-phase coupled to budding simultaneously. Although, the arrest and activation are mediated by the centrosome, there is likely a diffusible factor that prevents passing the DNA-licensing checkpoint and blocks re-entry into S-phase. How the parasite coordinates this arrest is a wide-open question for which no robust molecular leads are available.

## Outlook

In conclusion, the exotic varieties and flexibility in apicomplexan division modes originate in a compartmentalization of the cell cycle checkpoints which permit uncoupling of specific checkpoints from general cell cycle progression. The unconventional Cdks, Crks and cyclins making up the checkpoints engage with a unique and expanded set of ApiAP2 transcription factors that synergize into driving functional modules of the cell division process. Since the number of ApiAP2 factors correlates with genome size rather than division modes, the details of how the different division forms are wired is expected to be quite unique for each species and division mode, although the general wiring scheme is conserved across the division modes considered here. However, are there fundamental features that could have been missed that could explain some of the wiring mysteries? For example, do we have the full library of epigenetic DNA and histone modifications, which both seem to have unique features in the Apicomplexa. Another big unknown is the role of (long) non-coding RNAs (Li et al., 2020) which are speculated to be key epigenetic regulators (Kim, 2018). Besides the wiring of the modules, many questions remain regarding the modules themselves. For example, how does karyokinesis work, how do nuclei synchronize and through which mechanism? What is the basis for the differential control mother and daughter cytoskeleton stability? We hypothesize that many of these questions could be answered by comparative genomics of parasites with different division modes: across the division modes, particular modules are specifically amplified or combined, which permits the untangling of their contributions in the widely studied *Toxoplasma* endodyogeny and *Plasmodium* schizogony. In essence, this group of Apicomplexa are nature’s very own synthetic biology experiment and they provide an ideal set of organisms to unravel how the wiring of these different division modes is organized. Powerful genomic and genetic tools can now be applied on nearly any parasite system to tackle these questions.

## Author Contributions

M-JG, IC, and KE conceived the experiments, KE and IC performed the experiments, KZ and MD co-defined the division modules, the hierarchy and conservation of regulation, M-JG wrote the manuscript and all co-authors edited the manuscript. All authors contributed to the article and approved the submitted version.

## Funding

This study was supported by National Science Foundation (NSF) Major Research Instrumentation grant 1626072, a Knights Templar Eye Foundation Career Starter Award to KE, National Institute of Health grants AI128136, AI144856, AI110690, and AI152387 to M-JG, AI150090 to KZ and M-JG, AI138551 and AI153945 to MD, and AI117201 to IC. The funders had no role in study design, data collection and analysis, decision to publish, or preparation of the manuscript.

## Conflict of Interest

The authors declare that the research was conducted in the absence of any commercial or financial relationships that could be construed as a potential conflict of interest.
